# Multifunctional Organic Materials, Devices, and Mechanisms for Neuroscience, Neuromorphic Computing, and Bioelectronics

**DOI:** 10.1007/s40820-025-01756-7

**Published:** 2025-05-08

**Authors:** Felix L. Hoch, Qishen Wang, Kian-Guan Lim, Desmond K. Loke

**Affiliations:** 1 Faculty of Engineering, University of Southern Denmark, 5230 Odense, Denmark; 2 School of Integrated Circuits, Peking University, Beijing, 100871 People’s Republic of China; 3 Department of Science, Mathematics, and Technology, and the AI Mega Centre, Singapore University of Technology and Design, Singapore, 487372 Singapore

**Keywords:** Resistive switching mechanisms, Organic materials, Brain-inspired neuromorphic computing, Neuroscience, Neuromorphic bioelectronics

## Abstract

Neuromorphic computing has the potential to overcome limitations of traditional silicon technology in machine learning tasks. Recent advancements in large crossbar arrays and silicon-based asynchronous spiking neural networks have led to promising neuromorphic systems. However, developing compact parallel computing technology for integrating artificial neural networks into traditional hardware remains a challenge. Organic computational materials offer affordable, biocompatible neuromorphic devices with exceptional adjustability and energy-efficient switching. Here, the review investigates the advancements made in the development of organic neuromorphic devices. This review explores resistive switching mechanisms such as interface-regulated filament growth, molecular-electronic dynamics, nanowire-confined filament growth, and vacancy-assisted ion migration, while proposing methodologies to enhance state retention and conductance adjustment. The survey examines the challenges faced in implementing low-power neuromorphic computing, e.g., reducing device size and improving switching time. The review analyses the potential of these materials in adjustable, flexible, and low-power consumption applications, viz. biohybrid spiking circuits interacting with biological systems, systems that respond to specific events, robotics, intelligent agents, neuromorphic computing, neuromorphic bioelectronics, neuroscience, and other applications, and prospects of this technology.

## Highlights

- The review emphasizes the switching mechanisms of organic neuromorphic materials.
- In addition to these switching mechanisms, the capabilities of organic neuromorphic materials in tunable, conformable, and low-power applications, e.g., neuromorphic computing, neuroscience, and neuromorphic bioelectronics, are investigated.
- This review article offers a comprehensive examination of the capabilities of organic neuromorphic material-based devices to incorporate artificial intelligence into everyday activities.

## Graphical abstract

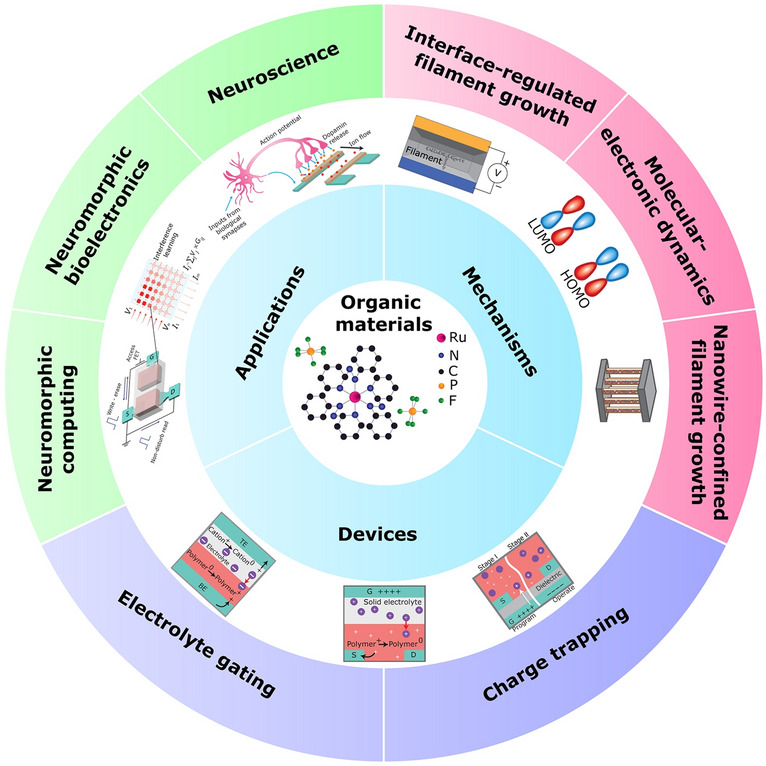

## Introduction

Deep learning and artificial intelligence algorithms are becoming increasingly essential in various applications, despite their similarities to the human brain. These algorithms are executed at a software level and rely on artificial neural networks, which are typically implemented on traditional von Neumann architecture computers [1–3]. The brain’s physical components, consisting of an interconnected and complex neuron system, function efficiently due to synapses’ chemical movements that control signal strength. The Hebbian learning principle posits that concurrently activated neurons create connections, serving as the foundation for memory and learning. This ability to adapt and change is primarily responsible for processing information within the brain, rendering it highly energy efficient compared to conventional computers, particularly in pattern classification and recognition.

Memristive devices, or memristors, are resistive switches with variable yet non-volatile electrical resistivity or optical reflectivity. These devices have been proven to exhibit synaptic functionality resembling Hebbian learning. They generate non-volatile memory arrays, perform basic pattern recognition task, and process information in hardware while consuming low energy. Researchers have demonstrated that neural network algorithms can be integrated into hardware, mimicking the brain’s efficiency and function on a small system [4–6].

To simulate the brain’s parallel operation, a memristive device-based network has to exhibit efficient parallel vector–matrix multiplication, symmetrical and linear programmable conductance states, and low energy consumption [7–9]. These features enable “blind” synaptic weight updates during learning. State-retention time requirements vary depending on the application, but longer times are generally preferred. For continuous learning scenarios, synaptic weights are regularly transferred to external memory, while for train-once inference-only applications, they are stored on-system for an extended period.

Bio-inspired systems using adjustable memory components have to replicate brain-like functions, e.g., long- and short-term potentiation, spike-rate-dependent plasticity, and spike-timing-dependent plasticity [10–12]. The neuromorphic devices’ requirements depend on the neural network architecture and application. To ensure optimal functioning, certain measurements are necessary for hardware-based neural networks, especially for neuromorphic devices relying on organic computational materials.

Tunable organic computational materials offer a promising alternative to traditional memristive materials in neuromorphic applications, particularly in online learning scenarios. These materials learn synaptic weights on systems, enabling real-time predictions. Organic neuromorphic materials offer innovative switching methodologies, maintaining a wider range of dynamic capabilities and low-energy operation, rendering them less stochastic [13–15].

Organic neuromorphic materials can be easily integrated into manufacturing methodologies, e.g., inkjet printing, and are cost-effective. They can be customized for specific applications through chemical synthesis. These materials have been utilized in biology applications and neuromorphic devices, potentially enabling flexible brain–computer interfaces [16–18]. Prior literature assessments in this field have been limited to organic computational materials and devices and often incorporating surveys that primarily investigates neuromorphic computing applications.

Herein, this review explores the resistive switching mechanisms that govern organic neuromorphic material usage, focusing on their ability to operate with low power consumption and to adjust conductance levels, and their potential for different applications. The review also explores prospective future paths, their lasting effects, and the challenges in implementing these ideas in real-world applications. The review is organized as follows: In Sect. 2, the resistive switching mechanisms, e.g., interface-regulated filament growth, molecular-electronic dynamics, nanowire-confined filament growth, and vacancy-assisted ion migration that underlie organic neuromorphic device operations, are examined. Section 3 investigates emerging organic neuromorphic devices. Section 4 provides a critical discussion of the prospects and obstacles for organic neuromorphic devices. Section 5 discusses the organic neuromorphic device’s biocompatibility and integration. Section 6 highlights the applications of organic neuromorphic devices, viz. neuromorphic computing, neuromorphic bioelectronics, neuroscience, and other applications. Section 7 ultimately concludes the review article.

## Mechanisms for Resistive Switching

The deterioration of non-volatile memory devices, e.g., flash memories, is a significant issue in commercial technology. Understanding their operational mechanisms and physical impact on device structure and performance is crucial for their market share. Organic neuromorphic device’s switching and failure processes are essential for optimizing their reliability and performance [19–21]. Organic neuromorphic devices utilize a resistive switching mechanism to dynamically adjust electrical conductivity or optical reflectivity, mimicking brain synapses, enabling memory and learning in artificial neural networks by adjusting conductance or reflectance levels via voltage or laser pulses. The organic neuromorphic devices’ reliability is linked to their fundamental operation principles, e.g., the availability and characteristics of primary species or particles involved, the extent of damage caused by each event, and whether switching is localized or area distributed. Disclosing reliability for organic neuromorphic devices is more complex due to the need for full clarification of the switching mechanism [22–24].

The process of evaluating a switching mechanism in organic neuromorphic devices involves a variety of analyses, e.g., analysing the dynamics of the state change and evaluating the temperature dependence of the electrical transport mechanism. Other techniques used include quantifying species concentrations using X-ray photoelectron spectroscopy, ultraviolet–visible spectroscopy, and in situ Raman spectroscopy, and assessing atom migration and morphology changes through cross-sectional transmission electron microscopy and backscattering scanning transmission electron microscopy, coupled with chemical analysis. Conductive atomic force microscopy and in-operando imaging are also utilized to address the deeply scaled area dependence of the switching mechanism. Physical simulations, viz. density functional theory and ab initio Monte Carlo simulations, are also utilized to analyse defect origins and diffusion or migration involved in the switching mechanism [25–27].

**Fig. 1:**
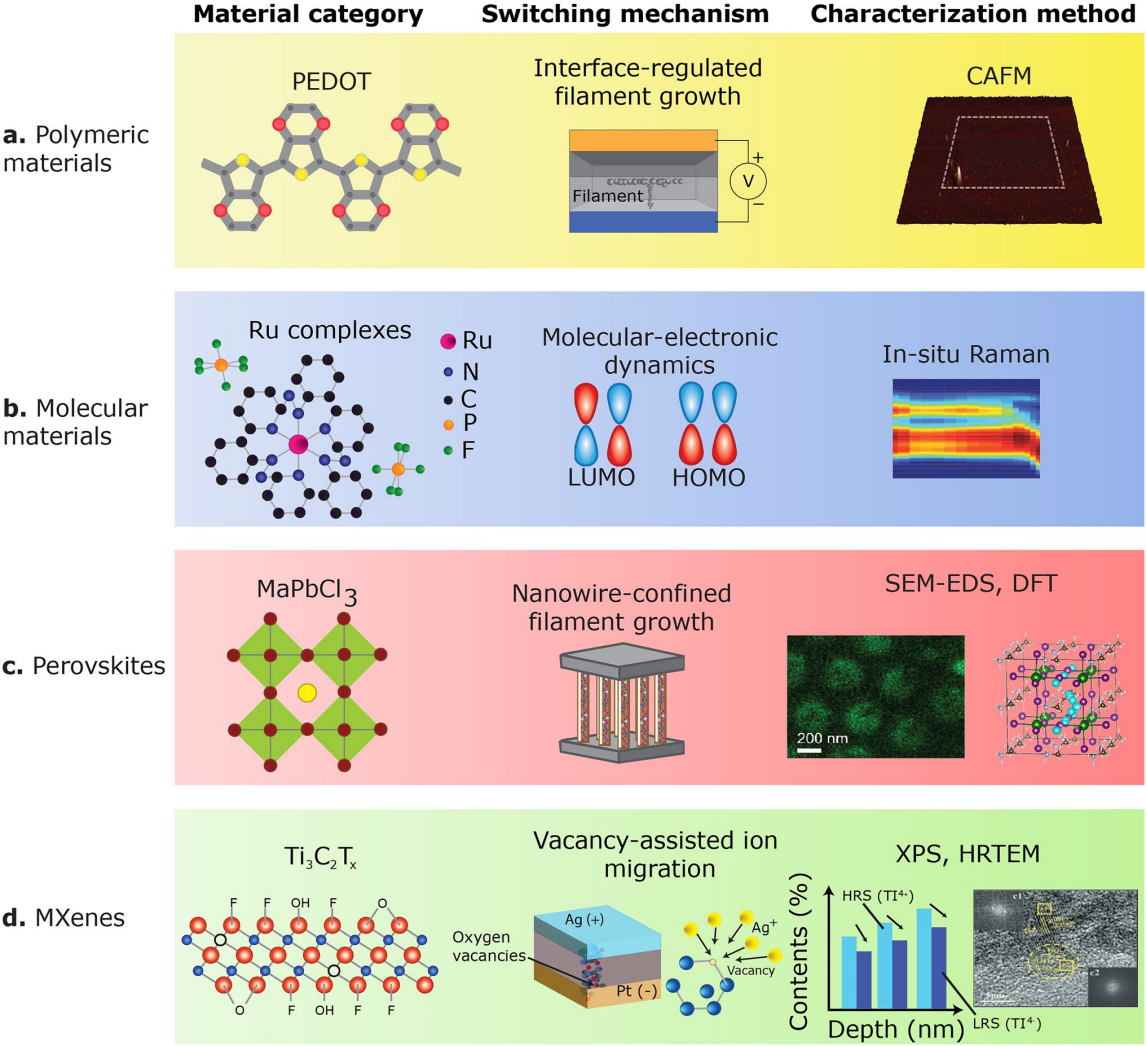
Evaluation of switching mechanisms in organic neuromorphic materials.** a–d** Overview of the proposed switching mechanisms and primary instruments employed in the investigation of neuromorphic materials. The utilized materials comprise **a** polymeric materials [28], Copyright 2020, Wiley–VCH GmbH; **b** molecular materials [29], Copyright 2020, Springer Nature; **c** perovskites [30], Copyright 2021, AAAS, and **d** MXenes [31], Copyright 2019, Wiley–VCH GmbH. Molecular-electronic dynamics or LUMO-HUMO resistive switching is a process where an electric field causes electrons to transition from the highest occupied molecular orbital (HOMO) to the lowest unoccupied molecular orbital (LUMO) within polymer chains, resulting in a change in the material’s electrical resistance and acting as a switch for electrical conductivity. Conductive atomic force microscopy (CAFM) is a technique used to analyse the electrical properties of polymeric materials at the nanoscale. In situ Raman spectroscopy is a method employed to analyse the chemical structure and characteristics of organic materials in real time, directly within the process or reaction environment. Scanning electron microscopy (SEM) combined with energy-dispersive X-ray spectroscopy (EDS) is a powerful tool for analysing the elemental composition and surface morphology of perovskite materials. Density functional theory (DFT) is a computational methodology utilized to investigate the optical, electronic, and structural properties of perovskite materials. X-ray photoelectron spectroscopy (XPS) is an essential methodology for examining the surface chemistry of MXene materials, offering comprehensive insights into surface terminations, oxidation states, and elemental composition, vital for comprehending their properties and prospective applications. High-resolution transmission electron microscopy (HR-TEM) is a crucial approach for investigating the intricate structure of MXene materials, revealing significant insights into their characteristics and morphology

This section explores the proposed switching mechanisms for organic neuromorphic materials (Fig. 1), their optimal electrical performance, and their implications for device reliability and performance. It also evaluates the impact of manufacturing processes, e.g., selector integration, lithographic patterning, and integration density, on switching behaviour. Table 1 provides a summary of various examples of switching processes in organic neuromorphic devices.

**Table 1:** Switching mechanism, device performance, and structure of innovative organic neuromorphic devices

Material	Switching mechanism	Patterning	Retention (s)	Switching time (ns)	Cycling endurance (number)	Cell size (µm^2^)	References
Au/PEDOT:PSS/PMMA/Ag	Interface-regulated filament growth	2 × 2 crossbars (shadow mask)	2.7 × 10^3^ at 300 K	200 × 10^3^	10^4^	2.5 × 10^5^	[28]
Au/[Ru(L)_2_]/(AuND)/ITO/YSZ	Molecular-electronic dynamics	Common bottom electrode (shadow mask)	10^6^ at 300 K	30	10^9^	Not reported	[29]
ITO/[Ru(L)_3_](PF_6_)_2_-(Au NPs)/ITO	Molecular-electronic dynamics	Common bottom electrode (shadow mask)	5 × 10^6^ at 350 K	Not reported	10^12^	9	[32]
Au:ITO/[Fe(L-)L_2_] PF_6_/Au	Molecular-electronic dynamics	16 × 4 crossbars (lithography)	2 × 10^5^ at 350 K	5 × 10^3^	Not reported	16	[33]
Ag/MAPbCl_3_ NWs/Al	Nanowire-confined filament growth	Common bottom electrode (shadow mask)	10^6^ at 440 K	0.3	4 × 10^6^	7.3 × 10^4^	[30]
Al/Ti_3_C_2_Tx/Pt/Ti	Vacancy-assisted ion migration	Common bottom electrode (shadow mask)	10^5^	200	10^6^	7.8 × 10^3^	[31]
Al/Ti_3_C_2_:Ag NPs/Pt	Vacancy-assisted ion migration	Common bottom electrode (shadow mask)	10^4^	2 × 10^3^	10^6^	6 × 10^3^	[34]

### Interface-Regulated Filament Growth

Area-distributed switching, influenced by charge or atomic rearrangement mechanisms, has been exhibited in multiple I-V cycles at the nanometre scale using conductive atomic force microscopy. The optimal electrical performance was examined on 9–16 µm^2^ devices. Encouraging findings reveal that polymeric layers maintain stability while designing top electrodes to conform to nanometre-scale crossbar arrays via electron beam lithography. However, comprehensive electrical performance characterization is required in traditional deeply scaled devices to validate their micrometre-scale equivalents.

Polymeric devices utilizing poly(3,4-ethylenedioythiophene) (PEDOT) (Fig. 1a), applied via drop casting, and silver electrodes depend on filamentary switching [28]. A polymeric device featuring self-compliance current was developed by incorporating an interfacial load polymer into a single device, functioning as an internal resistor to modulate the electric field, hence guaranteeing dependable and consistent conductive filament generation. The interfacial load polymer layer restricts the propagation of numerous conductive filaments (Fig. 1a), as demonstrated by conductive atomic force microscopy mapping of conductive regions and scanning electron microscopy analysis of the devices in the low-resistance state. Nevertheless, the potential for size reduction in conventional filamentary switching polymeric devices remains ambiguous, and their electrical performance is inferior to that of devices utilizing area-distributed switching.

### Molecular-Electronic Dynamics

Apart from polymeric devices that regulate electric field to limit conductive filament proliferation, molecular devices utilizing metal-azo-aromatics (Fig. 1b), e.g., Fe or Ru complexes, have demonstrated the highest neuromorphic device performance to date [29, 32, 33]. In these devices, the molecular layer is created via chemical reaction and precipitation and deposited using spin coating, resulting in thin layers with excellent surface roughness. The layers remain stable during electrode patterning and are compatible with the patterning process; however, this has yet to be verified for traditional molecular devices. A prevalent fundamental switching mechanism involves the modification of the redox states of ligands inside the metal complex (Fig. 1b), facilitated by the movement of counterions. The switching dynamics are corroborated by ultraviolet–visible spectroscopy, in situ Raman spectroscopy, and density functional theory. The integration of these methodologies facilitates the correlation of the distinctly observable redox peaks at various applied voltages with the population of molecular orbitals in the metal-azo, as determined by density functional theory calculations.

The most effective molecular devices for neuromorphic performance metrics documented to date are those utilizing metal-azo aromatics, specifically iron or ruthenium complexes. These devices exhibit the potential for enhanced complexity in their neuromorphic properties, ranging from non-volatile bipolar resistive switching to voltage-dependent multilevel non-volatile characteristics, as well as parallel digital adders and more intricate decision trees in device arrays facilitated by deterministic multi-threshold switching events.

### Nanowire-Confined Filament Growth

In addition to molecular devices that show molecular-electron dynamics, perovskite-based devices, e.g., MAPbCl_3_ nanowire computational devices (Fig. 1c), exhibit significant promise for neuromorphic computing applications, owing to their long state-retention time (10^6^ s at 300 K), high cycling endurance (> 10^6^), and rapid switching speeds (~ 300 ps) [30]. The devices were characterized at various temperatures, benefiting from their stability following epoxy encapsulation. The migration of Ag^+^ from the Ag electrodes predominates the switching mechanism (Fig. 1c), with elevated concentration of Ag observed in the low-resistance state. The diffusion barriers for Ag can be optimized by selecting Cl, Br, or I in the lead halide perovskite’s composition, as established by density functional theory analysis.

Silver atoms facilitate highly effective resistive switching in MAPbBr_3_ and MAPbCl_3_ perovskite-nanowire neuromorphic devices featuring aluminium and silver electrodes. The researchers employed scanning electron microscopy in conjunction with energy-dispersive X-ray spectroscopy to investigate the presence of Ag within the nanowires, demonstrating unequivocal evidence of Ag within the nanowires in the low-resistance state. Density functional theory was employed to compute the energy landscape for the silver atoms’ diffusion and to evaluate various state-retention properties.

These devices have potential computational performance for neuromorphic computing applications, demonstrating endurance comparable to that of commercially available solutions, multiple approaches investigated for enhancement, and outstanding retention assessment. Recent studies have revealed that traditional halide perovskites are vulnerable to environmental influences, e.g., oxygen and moisture, resulting in a decline in their neuromorphic performance. Enhancing the halide perovskites’ stability during photolithography is essential to unlock the complete potential of these materials in neuromorphic devices.

### Vacancy-Assisted Ion Migration

Besides perovskite materials that display nanowire-confined filament growth, Ti_3_C_2_T_x_, a conductive MXene (Fig. 1d), was synthesized using chemical etching or oxidation followed by exfoliation to generate the most efficient organic MXene neuromorphic devices [31, 34]. Titanium vacancies and oxygen vacancies from readily oxidized titanium within the MXene significantly influence the resistive switching process (Fig. 1d). High-resolution transmission electron microscopy and X-ray photoelectron spectroscopy revealed fluctuations in TiO_x_ peaks between high- and low-resistance states, elucidating the switching behaviour. Downscaling has not been evidenced in traditional examples; however, it may be viable owing to recent advancements in MXene stability during photolithography. The Ti/O vacancies’ migration is a spatially dispersed phenomena, enabling neuromorphic properties at reduced dimensions. Nevertheless, the usage of Ti_3_C_2_T_x_ as a switching layer in conventional neuromorphic devices may be constrained, and the diminished conductivity in the high-resistance state may be attributed to significant oxidation of the titanium domains.

## Cutting-Edge Organic Neuromorphic Devices

Organic computational devices, designed for neuromorphic computing applications, comprise a two-terminal metal–insulator–metal arrangement that exhibits two switchable and stable resistance states [17, 35–38]. They consist of various dynamic materials, e.g., ferroelectric layers, donor–acceptor complexes, small molecules, and polymers. Resistive switching in organic neuromorphic materials occurs through physical mechanisms, viz. electromigration, ionic charge transfer, and localized conduction. The main focus will be on organic neuromorphic devices that allow for continuous resistance tuning, which is well-suited for on-system inference and learning using parallel multiply-accumulate operations. The similarity between biological synapses and organic neuromorphic devices is important for replicating the biological neural networks’ behaviour using artificial synapses.

**Fig. 2:**
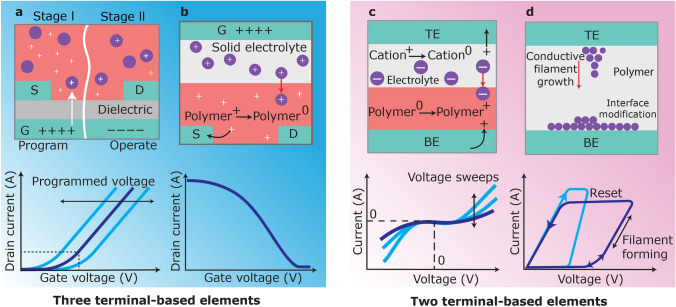
Analysis of resistive switching in organic neuromorphic devices. **a** In stage I, viz. Program, a high potential transfer charges to the nanoparticles in the film. In stage II, i.e. Operate, a reduced voltage on the gate is employed to activate the channel. The charge amount in the film determines the turn-on potential. D represents drain, S signifies source, and G denotes gate. **b** Organic electrochemical redox switching. An applied voltage on the gate induces ion migration from the electrolyte into the polymer, thereby altering its redox state and conductivity. **c** Two-terminal redox switching involves a counter-redox process. Upon the application of input voltage, a redox reaction transpires in both the polymer and the solid electrolyte, leading to altered conductance. BE signifies the bottom electrode and TE denotes the top electrode. **d** A two-terminal organic neuromorphic device utilizing bias-dependent interface alteration, as well as the growth of localized conductive filaments or alternative conductive pathways. The lower chart illustrates the current–voltage characteristic of a filament-forming device

Neuromorphic computing and memory applications display different criteria, with neuromorphic computing requiring stringent state-retention time, while high-speed memory applications require high cycling endurance and fast switching speeds. Neuromorphic devices require a wide range of distinct resistance states to effectively perform neural network functions. Various neuromorphic devices have been developed using distinct resistance tuning mechanisms found in organic computational materials and devices. Resistive switching in most devices is based on charge trapping or electrochemical doping (Fig. 2) [39, 40].

### Resistive Switching Based on Charge Trapping

Researchers have developed a new mechanism for demonstrating neuromorphic capability and memory effect in organic field-effect transistors using charge trapping (Fig. 2a). These devices depend on charge storage within non-metallic or metallic nanoparticles that function as nanoscale capacitors embedded in organic semiconductors, e.g., poly(methyl methacrylate) or pentacene. The presence of charged particles alters drain–source behaviour, allowing for resistance tuning [41–45].

The new device’s mechanism differs from traditional organic neuromorphic devices or floating-gate transistors due to nanoparticles embedded inside the material. However, a gate potential is still needed for read operation. A pseudo-two-terminal setup was created, requiring only a drain-source voltage, allowing channel conductance to be modified based on pulse frequency.

Organic neuromorphic transistors with charge-trapping properties have potential for low power computing due to extended state-retention durations and high channel resistance. However, traditional narrow channels’ capacity for nanoparticles may limit device efficiency in densely packed arrays. Researchers have yet to determine whether conventional charge-trapping devices can be rendered smaller while maintaining consistent performance and low operating noise.

### Redox-Based Switching Controlled by Electrolyte Gating

Electrochemical doping can be utilized to adjust the device’s resistance by using a gate electrode to introduce or remove ions from the organic film (Fig. 2b, c). This technique alters the film’s doping, i.e. the redox state. Electrolyte-gated conducting polymers were used to demonstrate various switching behaviours, and other nanowires, electrolytes, and polymeric materials have been employed in similar configurations. These devices typically display two resistivity states: high and low, but can be continuously adjusted by modifying the pulse frequency or gate potential, allowing for more than two resistance levels [36, 46–49].

Researchers have modified an organic electrochemical transistor, incorporating a de-doped-conducting polymer channel and conducting polymeric gate. This design resembles an organic battery, maintaining electrical neutrality through a counter-redox reaction that improves state retention. Traditional battery-based devices utilize solid electrolytes and polymers, but lack an ionic-conductor separation layer, leading to limited state retention and unwanted self-discharge.

The adjustable resistivity and customizable properties of electrolyte-gated organic computational materials and topologies have exhibited significant potential for neuromorphic computing applications.

## Organic Device Possibilities and Limitations

**Fig. 3:**
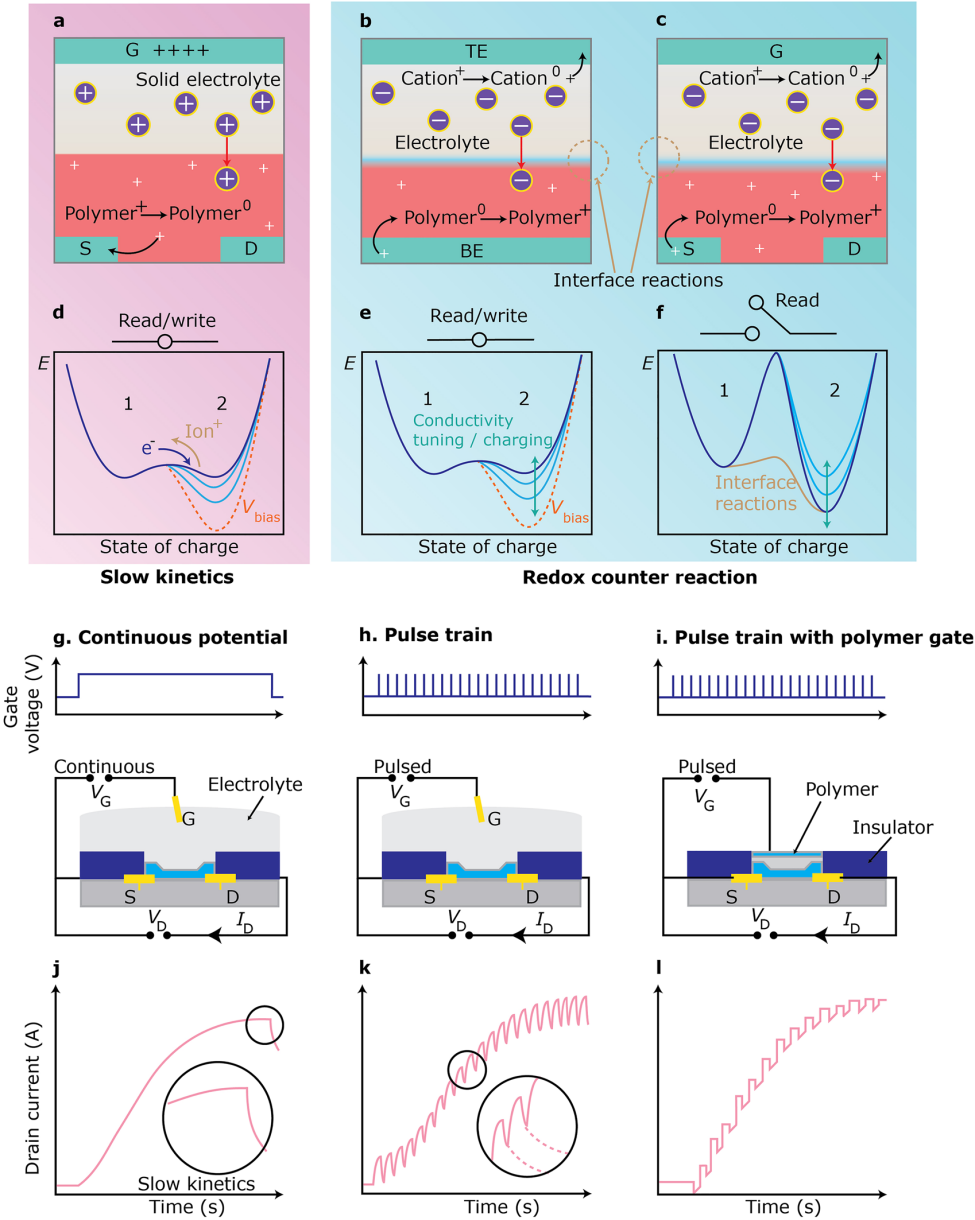
Stability and conductance tuning methodologies in organic neuromorphic devices. **a** Electrochemical transistor-based devices depend on slow kinetics to maintain a conductance state. Counter-redox reaction-based devices depend on a counter reaction within the electrolyte in **b** a two-terminal configuration and **c** a three-terminal gated configuration to maintain electrical neutrality and improve state retention. **d–f** Correlation between energy and charge state. **d** In electrochemical transistors, a bias alters the electrochemical potential of the polymer films, viz. the dashed red line, but provides minimal stability due to the low energy barrier for self-discharge. **e** In two-terminal counter-redox reaction mechanisms, the writing and subsequent reading processes resemble those of ordinary electrochemical transistors; however, state retention is improved in the absence of applied voltage. **f** In three-terminal gated counter-redox reaction-based devices, the read operation is independent of the write operation, hence inhibiting self-discharge through a substantial energy barrier. Nevertheless, in the absence of adequate separation of the electrodes participating in the redox reaction, e.g., through an electrolyte, undesirable interfacial reactions, i.e. the dashed orange line, diminish the barrier for localized self-discharge. Depictions of the electrochemical devices using **g** continuous potential, **h** pulse train, and **i** pulse train with a polymeric gate. **j** A continuous gate potential progressively alters the conductance, which reverts to its initial value upon the removal of the gate potential. **k** A pulse train. Brief pulses analogous to those in **j** lead to the incremental alteration of conductance. Upon cessation of the pulses, the conductance reverts to its initial level. **l** The pulse train utilizing a polymeric electrode gate and the separation of write and read operations ensures that the attained conductance condition persists after the termination of gate pulses

Organic computational materials have exhibited potential for neuromorphic computing applications due to their diverse properties, e.g., low energy requirements and tunable conductivity. These materials can be easily customized electrically, mechanically, and chemically to meet specific requirements. Despite their potential, challenges remain in terms of device variability, array integration, and the materials and devices’ stability. Despite recent literature addressing specific issues, several obstacles persist in the field as organic computational materials continue to evolve. This has led to a diverse range of organic memory and neuromorphic devices (Fig. 3) [50–53].

### Short-Term Plasticity

The brain’s neuronal transmission occurs over time and is dynamic, with synaptic plasticity determining changes in communication strength. Long-term effects are associated with memory and learning processes, while short-term plasticity allows for computing capabilities. These features are observed in organic neuromorphic devices, with differences based on their intended purpose [54–56]. Short-term plasticity is utilized to demonstrate synaptic capability, e.g., short-to-long-term plasticity, spike-rate-dependent plasticity, and spike-timing-dependent plasticity, and to emulate synapses. Artificial synapses, viz. devices that mimic these synaptic functions, are valuable in investigating spiking neural networks and brain inner workings. They incorporate leaky integrate-and-fire components, emulate biological neurons’ function by regulating signal transmission to neural networks’ subsequent layers, and exhibit conductance determined by prior pulses. This process involves integrating incoming signals until a specific threshold is reached, allowing the neuron to emit a signal.

Spike-timing-dependent plasticity is a crucial process in biological neural networks that regulates signal intensity between two neurons within a synapse. It relies on the temporal discrepancy between post- and pre-synaptic pulses at the synapse, with a decrease in the time gap leading to increased synaptic weight modulation. Spike-timing-dependent plasticity is a local learning mechanism, unlike error backpropagation, which applies to array operations. It has been demonstrated on the single device level in two-terminal organic neuromorphic devices, electrolyte-gated three-terminal devices, and charge-trapping devices. Spike-rate-dependent plasticity, closely related to spike-timing-dependent plasticity, establishes a correlation between pulse frequency and weight modulation. Short-to-long-term plasticity refers to lasting conductance changes due to repeated stimulation [13, 27, 57–59].

Research on artificial synapses is advancing the replication of biological synapses’ functions, particularly in spiking neural networks. Efficient on-system learning requires increased state-retention times in organic neuromorphic devices and a global learning architecture. Artificial synapse devices with “long-term memory” facilitate the achievement of these objectives, enhancing the understanding of biological neural networks.

### Long-Term Plasticity and State Retention

Artificial neural networks and vector matrix multiplication benefit from improved stability and long state-retention times [60, 61]. These times were first observed in non-volatile organic memory, where maintaining state stability is crucial to avoid data loss. Recent demonstrations reveal that solution-processed azo aromatics memory devices have retention periods of eleven days, while multilevel optical memory devices display retention lengths of up to five hundred days. Electrochemical doping allows neuromorphic devices to operate within a wider number of states and conductivity range. Enhancing state-retention capability is crucial for efficient usage in hardware-based neuromorphic arrays.

Depletion mode is the operational mode of conducting polymer-based organic electrochemical transistors, where the channel remains highly conductive until ions are introduced, leading to mobile holes’ removal and conjugated polymer backbones’ de-doping. When the gate voltage is deactivated, ions return to the electrolyte, and the polymer undergoes doping again (Fig. 3a, d). This phenomenon was initially utilized to demonstrate short-term plasticity capabilities in a PEDOT:PSS-based electrochemical transistor. Traditional electrochemical transistor’s state retention relied on the ions’ slow passage, rendering it volatile. However, by modifying the organic materials, a non-volatile behaviour, attributed to the polymer’s structural rearrangement, was demonstrated [62–64]. To enhance state retention, researchers have created a non-volatile memory effect in organic PEDOT-based electrochemical transistors using a specific current biasing strategy. The incorporation of poly(vinylidenefluoride-co-trifluoroethylene) (P(VDF-TrFE)) ferroelectric layers generates a persistent memory effect, typical for ferroelectric materials. Lowering the conductive channel’s length down to 20 µm extended the retention period of polyaniline-based electrochemical transistors to 10^3^ s and improved switching speeds. Restricting ion-dopant movement in the redox process also increased their retention duration. However, this results in electrochemically metastable films due to traditional polymers’ trapped ionic charges.

A two-terminal electrochemical device using ethylviologen diperchlorate (EV(ClO_4_)) embedded in a solid polyethylene oxide electrolyte on a triphenylamine-containing polymer (BTPA-F) surface is also utilized to maintain electrical neutrality across all layers of the device’s film to improve state retention. This counter-redox reaction allows the cation in the solid electrolyte to accept an electron, while the polymer side withdraws an electron, creating a mobile hole (Fig. 3b, e). This device functions as an electrochemical battery with mobile ions and two redox components. However, each pulse between the top and bottom electrodes causes bias throughout the redox component, disrupting traditional devices. Despite being electrically neutral, conventional device lacks ultra-high stability, rendering it susceptible to volatility during read operations [65–68].

Researchers have also developed a methodology to isolate redox reactions during conductance modulation, viz. Write (Fig. 3c, e), from conduction-state assessment in a polymer film, i.e. Read (Fig. 3c, f), using a three-terminal electrochemical configuration and polythiophene as the conducting polymer for enhancing state retention. This approach improved stability in the two-state device and allowed for better differentiation between states. The modulation results from recombination reactions at the interface between the two sides of the redox component. By introducing a charge-separation electrolyte layer, a device similar to an electrochemical battery was created, which allowed ion movement, preventing interface recombination reactions and improving state retention for multiple states.

To improve state retention, conducting polymers can be adjusted to different reduction and oxidation states using electrochemical doping. However, further reduction renders traditional conducting polymers more susceptible to oxygen doping, limiting their ability to retain their state. This limitation can be mitigated by adjusting the polymer’s level to stabilize its reduced state or by using encapsulation techniques to minimize oxygen doping impact.

Besides electrochemical doping, charge trapping has been utilized to enhance state retention. Charge trapping is a metastable methodology used in gold (Au) nanoparticle-pentacene neuromorphic devices with a retention time exceeding 10^3^ s. The gold nanoparticles are located within a polymeric channel, allowing electronic charge conduction. However, this arrangement limits traditional devices’ retention and stability. Nevertheless, researchers utilized a single layer of TEDOT-modified gold nanoparticles, resulting in an increased retention period of 10^5^ s [69, 70].

### Number of Conductance States

Traditional organic and inorganic devices exhibit a high conductivity state and a single low conductivity state. However, memristive devices, e.g., conductive filament-based devices, vacancy migration-based devices, and phase-change based devices, demonstrate a greater number of programmable states. Nevertheless, conventional memristive devices exhibit unpredictability and disclose substantial write noise. Biological synapses continuously adjust their synaptic weight and conductance. Hardware-implemented forward-inference neural networks require analogue variation in organic synaptic devices, which facilitates analogue computing and improves artificial neural networks’ accuracy.

Organic neuromorphic devices exhibit a wide range of conductance states by adjusting the charge on gold nanoparticles distributed across the transistor channel [71–75]. By using repulsion between mobile charge carriers and charged nanoparticles, precise control over channel conductance was achieved, resulting in a 10^3^–10^4^ on/off ratio. Traditional organic neuromorphic devices achieve multilevel storage by charging a separate gate electrode, similar to multilevel floating-gate memory, but require elevated write voltages.

Redox coupling mechanisms involving electrolytes rely on the gate potential to adjust channel conductance (Fig. 3g, j) [76–78]. Several mechanisms demonstrate that a train of pulses reduce or enhance channel activity (Fig. 3h, k). These tuning processes depend on the polymer’s redox state, caused by the gate electrode’s electrochemical potential. Modifying the redox state leads to conductivity changes by increasing or reducing the quantity of mobile charge carriers. Charge transport can be effectively regulated by adjusting the pulse frequency and gate potential, similar to biological synapses. The channel’s ultimate conductance remains unaffected by modulation through continuous gate potential or short pulses.

An organic neuromorphic device has been developed that accurately adjusts channel conductivity using polyethylene-imine-polymer protonic doping (Fig. 3i, l). This device operates similarly to an organic battery, with both polymer electrodes changing their redox state during switching. To improve state retention and prevent charge exchange, the electrodes are electrically isolated during non-writing and reading periods. This results in a non-volatile neuromorphic device that precisely adjust conductance throughout the entire spectrum of polymer redox states, similar to conductivity regulation through n- or p-type silicon doping [79–81].

Conventional two-terminal organic devices exhibit two conductance states, but several multi-state organic neuromorphic devices exist. Researchers have used a Ti:PEDOT:PSS:Ti sandwich structure to achieve one hundred different conductance states in a metal-polymer-metal configuration [82, 83]. This modification allows the Ti compound to grow and migrate, resulting in a progressive conductance shift. To effectively utilize these two-terminal-based concepts, write noise has to be minimized during random switching. This is crucial for integration into crossbar arrays, facilitating operation, and reducing dimensions.

Researchers have developed a multi-state organic neuromorphic device using optical manipulation of charge transport within a poly(3-hexylthiophene) (P3HT) film mixed with photochromic diarylethene [84–87]. By exposing the polymer matrix to green and ultraviolet light irradiation, the energy levels of the photochromic material were adjusted to match those of P3HT, resulting in an improved charge transport. This presents an opportunity for advancing light-assisted programming and neuromorphic applications, which addresses limitations in traditional memristive-based crossbar arrays without complex access devices.

### Energy Consumption

The energy consumption per synapse in organic devices is closely linked to read stability, particularly in two-terminal organic neuromorphic devices. Low switching energies reduce stability, since the process of reading a state disturbs it. To address this, elevated voltages are used for writing to a new state, while reduced voltages are utilized for reading the current state [88–90]. Conventional inorganic memristive devices display writing energies ranging from 0.1 to 1,000 pJ, with device diameters ranging from 0.1 to 5.0 µm. To compete with traditional complementary metal-oxide semiconductor logic circuits, switching energy needs to be below 1.0 pJ.

Energy losses in electrode lines connecting traditional organic neuromorphic devices in an array require a specific minimum voltage for the core device, which can be high depending on the switching mechanism. Excessive resistive losses lead to increased energy expenses. Conventional filament-forming neuromorphic devices exhibit a significant difference in energy between depression and potentiation due to high resetting current. A polymer-based filament formation process requires a 0.1–100 pJ energy range to switch, depending on factors, e.g., conductance change and pulse number.

Redox coupling in polymeric materials exhibit a low activation energy due to the presence of free volume spaces between polymer chains. This allows for reversible and inexpensive ion exchange, resulting in low energy switching. The energy required for a full write–read–erase operation of a polymer device is less than 10 pJ, while the energy for a single spiking operation for short-term modulation is approximately 10 pJ.

Recent studies identified a hybrid inorganic–organic perovskite neuromorphic device with low switching energies, comparable to 10 femtojoules per synaptic event in the brain. This reduced energy is associated with diminished state stability, suggesting a reversible switching process. The ion’s sluggish movement restores the initial state, but further investigation is needed to validate this [91–95]. The energy needed to switch a 1,000 µm^2^ device in a battery-like artificial synapse with improved stability is less than 10 pJ, achieved by tuning the voltage source to the device’s open-circuit potential using a potentiostat. The energy is directly proportional to the channel size, resulting in a 35 attojoule minimum switching energy for a 300 nm × 300 nm device. This energy difference results from improved state retention and a minimal charge applied to the capacitor, allowing for discrete conductance states with a narrower conductance difference.

Traditional charge-trapping devices function at elevated voltages, resulting in increased switching energies. However, two-terminal bi-stable organic neuromorphic devices operate at reduced voltages. A recent investigation revealed that improving the fabrication process of a nanoparticle organic field-effect transistor decreased the required switching voltage to 1.0 V and the corresponding energy to 2.0 nJ [96–100].

Organic neuromorphic devices significantly reduce the energy needed for programming by decreasing their dimension. The minimum energy required for an electrolyte-gated polyethylene oxide nanowire device is approximately 1.0 femtojoule. Organic computational devices display potential for low-power neuromorphic computing. However, the energy consumption of the entire neuromorphic system is crucial. A traditional low-power device that cannot be integrated into an energy-efficient system lacks significance. Therefore, careful consideration of individual device efficacy and integration is essential when building a neuromorphic system.

### Cycling Endurance

Neuromorphic arrays need to be investigated for their long-term usefulness, especially during extensive write-read cycles [101–103]. However, the exact endurance requirements remain uncertain. Dynamic and static random-access memory can be cycled over 10^16^ times, while non-volatile flash memory exhibits an approximately 10^4^-cycle lifespan. Endurance measurements have been conducted by cycling across the entire dynamic range or between neighbouring conductance states. Successful cycling has been shown in various devices, with a range of 20 cycles in two-terminal Ti/PEDOT:PSS/Ti electrochemical devices to 800 cycles in TEDOT-modified gold nanoparticle charge-trapping devices. Research reveals that polyaniline–polyethylene oxide redox-gated electrochemical devices exhibit 50 cycles, but subsequent investigations reveal that the switching endurance can be increased to 10,000 cycles by utilizing micrometre-scale polyaniline active-channel lengths. Optical poly(3-hexylthiophene)-photochromic diarylethene neuromorphic-based devices demonstrate 10^7^ s state-retention times, with minimal degradation even after 70 cycles.

## Biocompatibility and Integration

**Fig. 4:**
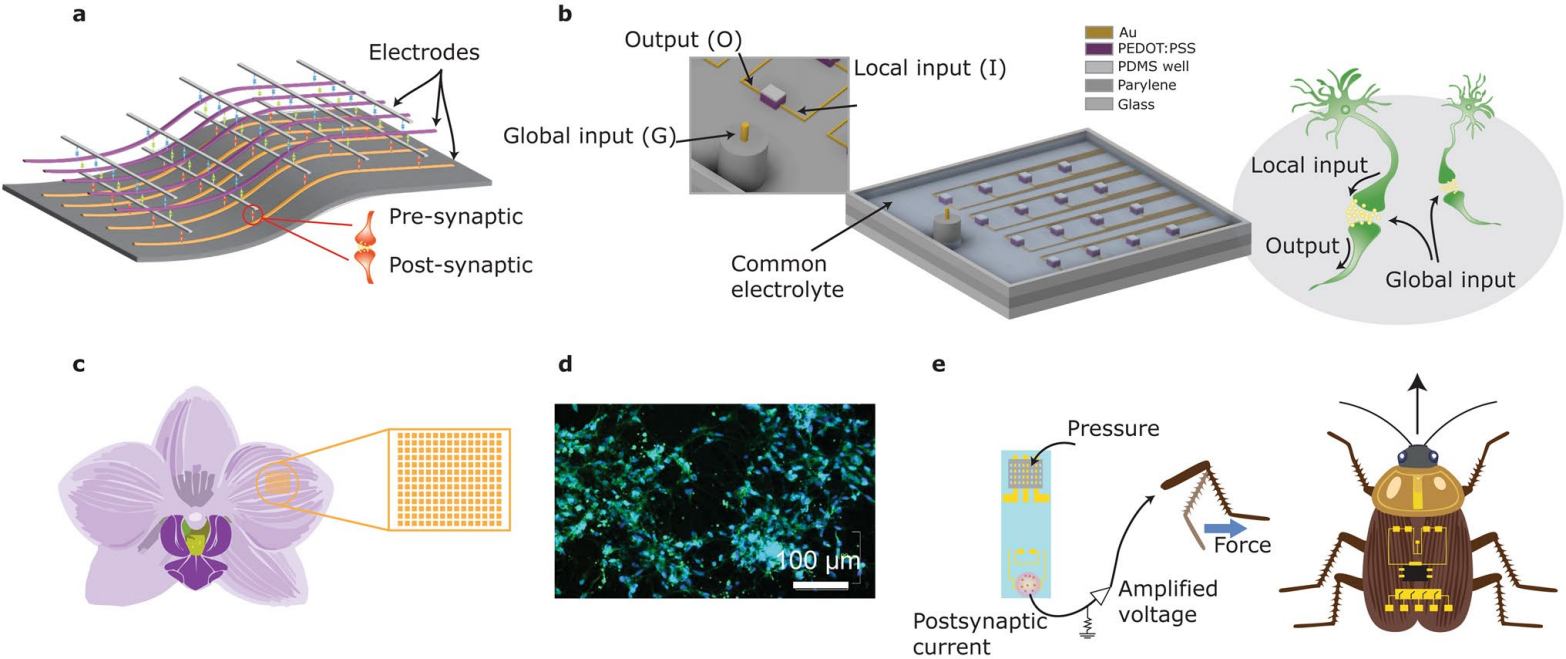
Illustrations of functionality and integration.** a** A three-dimensional flexible synaptic array. **b** Global gate-induced impacts on an organic neuromorphic array. **c** An adaptable organic-array implant structure adheres to a flower petal’s surface. **d** Neurons positioned atop an organic neuromorphic array [104], Copyright 2016, Elsevier. **e** Artificial flexible afferent nerve linked to biological nerves in an insect

Traditional organic neuromorphic devices face challenges in large-scale integration due to low device yield and reproducibility. However, recent advancements have shown successful implementation of integrated devices, e.g., the extensive integration of organic neuromorphic devices within a flexible three-dimensional network design (Fig. 4a). This network consisted of three superimposed copper-doped polymeric layers, functioning as a non-volatile neuromorphic array. Correlated learning was demonstrated in single artificial synapses integrated with a selector device using a spike-timing-dependent plasticity process.

In addition to extensive arrays, other implementations of functioning organic neuromorphic circuits have been documented, including a single-layer perceptron comprising two or three organic neuromorphic devices capable of classifying inputs through supervised learning. The dual-device architecture incorporated an activation function using two organic resistors and an organic field-effect transistor, but the training of the neuromorphic devices was conducted offline. On-chip training was used to execute NOR and NAND functions through basic threshold currents, as well as through intricate circuitry that integrated organic neuromorphic devices with CMOS-based neurons.

The electrolyte is a crucial characteristic of organic redox-based neuromorphic devices, which facilitates the design of innovative structures. An example is an architecture consisting of a single channel and several gates, allowing the spatiotemporal integration of applied signals at the gates to regulate the channel’s state. The electrolyte connects a single gate to numerous channels, akin to the global regulation of neurons in the brain (Fig. 4b). This characteristic is advantages in extensive neuromorphic arrays requiring a shared bias.

Organic materials’ biocompatibility and pliable mechanics render organic neuromorphic devices and arrays attractive for biological interfacing. These devices have been utilized in several applications, encompassing bio-inspired neuromorphic devices, biomimetics, biosensors, and implants (Fig. 4c). Synaptic operation and short-term potentiation in organic neuromorphic arrays demonstrate their capability to function with biological cells positioned above them (Fig. 4d). Recently, organic ion gel-gated transistors were employed in a flexible artificial afferent neuron for neuroprosthetics and neurorobotics (Fig. 4e). Further development improves the relationship between synaptic neuromorphic arrays and the adaptative regulation of organ, tissue, and cellular physiology and functions, enhancing site-specific sensing and monitoring.

## Applications

**Fig. 5:**
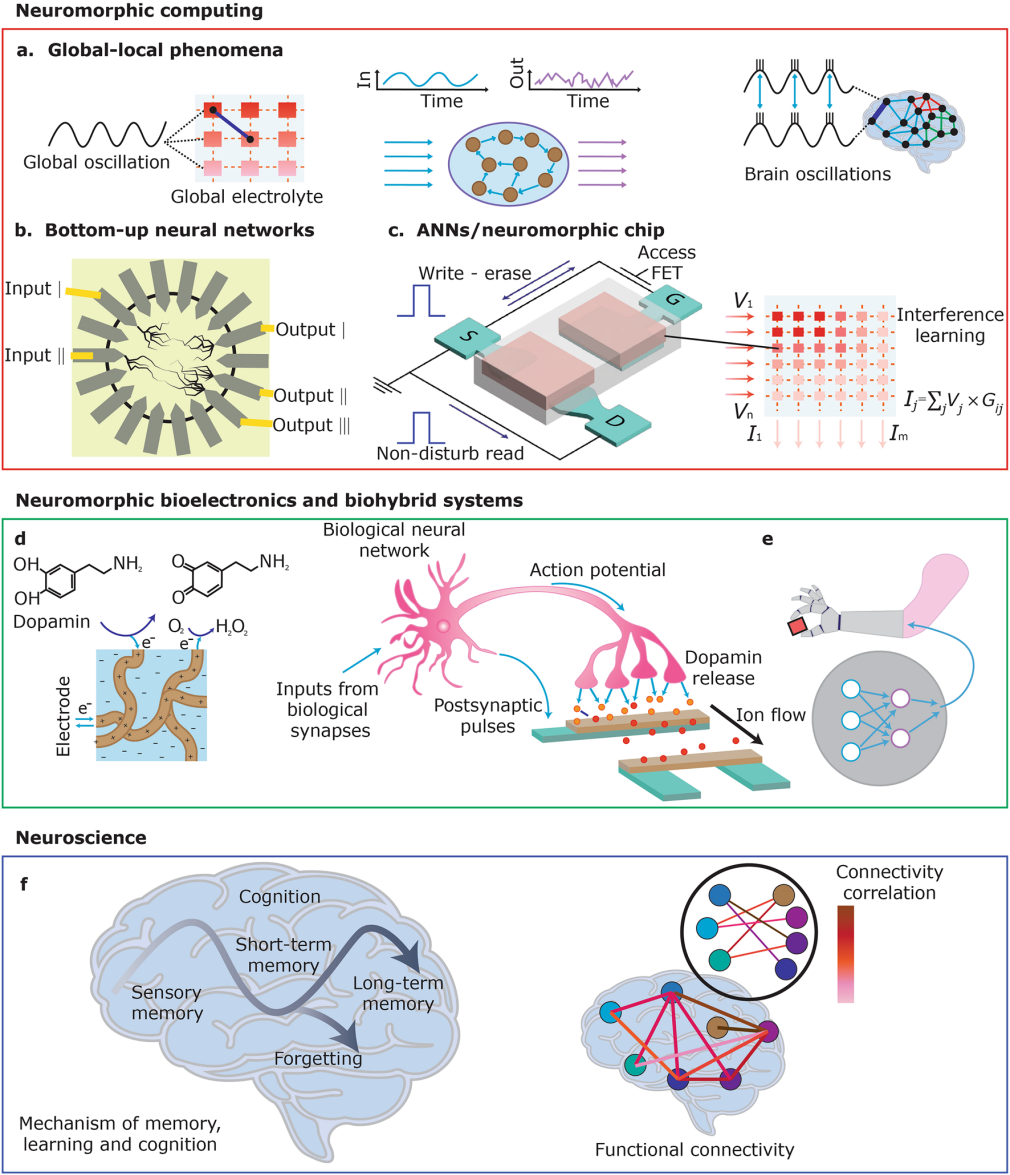
From devices to integrated networks and frameworks, and biohybrid materials and systems.** a** A global electrolyte common to devices facilitates a functional link akin to the brain’s global oscillations. **b** Bottom-up dendritic structures of conducting polymers can be achieved using electropolymerization in a solution containing monomers. These intricate structures function as a reservoir and transform nonlinear inputs into higher dimensional computational spaces, facilitating in materio computing, such as classification. **c** Organic artificial synapse serves as a fundamental component for ANNs/organic neuromorphic systems. The organic neuromorphic systems facilitate local or distributed learning and inference. **d** An organic artificial synapse is capable of processing both biochemical signals, viz. neurotransmitters, and co-current electrical signals, i.e. action potentials. **e** Biohybrid artificial nerve systems replicate the neural signal transmission process, which involves the conversion of biochemical signals into electrical signals by the brain. These signals are then transmitted by the artificial nerve to power prosthetics. The conversion, recognition, and transmission processes are facilitated and mediated by multimodal neural networks, which are composed of biochemical and electrical components. **f** High-performance and scalable flexible organic neuromorphic devices for brain–computer interfaces enhance neuroscience studies by facilitating long-term stable, large-scale, high-resolution brain mapping

Organic computational devices, along with their associated devices and networks, have the potential to develop innovative neuromorphic architectures and applications (Fig. 5).

### Neuromorphic Computing

The integration of multiple organic computational material-based neuromorphic devices through an electrolyte has been used for achieving a new paradigm in neuromorphic computing, wherein local processes, e.g., synaptic plasticity, and global control enable the execution of biologically-realistic neuromorphic capabilities and effective learning algorithms (Fig. 5a). This facilitates concurrent electrochemical interactions among devices, permitting wireless ‘soft’ linkages. A global electrochemical oscillation in the common electrolyte synchronizes multiple devices, akin to the way brain oscillations coordinate neural population activity. This global–local interaction allows various neuromorphic architectures, permitting dynamic and flexible manipulation of the structural network’s topology. For example, researchers have shown that the outputs of PEDOT:PSS-based organic neuromorphic devices can be synchronized by a global oscillatory input, despite individual inputs being stochastic and independent. This temporal coupling occurs at a specific phase of the global oscillation, analogous to the phase locking of neurons to brain oscillations [105]. In another instance, a bio-inspired iontronic multiplexer, using spatiotemporal dynamics of organic electrochemical transistors and an electrolyte functioning as the common communication medium, was investigated. The iontronic system discriminates locally random-access events without requiring peripheral circuitry or address allocation, thereby significantly decreasing integration complexity [106].

Neuromorphic functions utilizing organic computational materials can also be achieved at both individual device and network levels through bottom-up methods, viz. electropolymerization (Fig. 5b) [41, 107]. A method for PEDOT electropolymerization was established, enabling the formation of fibres with regulated lengths and diameters. This methodology was then adapted to grow “evolvable” organic electrochemical transistors, emulating synaptogenesis. The time constants of synaptic plasticity in electropolymerized structures can be adjusted through growth conditions, facilitating learning across timescales ranging from milliseconds to hours. Chemical polymerization facilitated the in vivo synthesis of chlorinated polyethylene fibres, serving as bioelectronic interfaces and soft substrate-free conducting materials inside the biological milieu through metabolite-driven polymerization. Evolvable networks have been extended to encompass entire neural networks, including the development of organic pattern classifiers and nonlinear random neural networks for real-time categorization of bio-signals. For instance, PEDOT can be precisely adjusted post-microfabrication via the electropolymerization method. This technique utilizes both transconductance and impedance, which can be customized to fulfil various sensing requirements. Material studies utilizing Raman spectroscopy, atomic force microscopy, and scanning electron microscopy demonstrate that electropolymerization enables meticulous regulation of PEDOT microdomain organization, thereby affecting the iono-electronic characteristics of organic electrochemical transistors. The volumetric capacitance and effective mobility of PEDOT/polystyrene sulfonate significantly influence the transconductance and impedance of organic electrochemical transistors. This approach exhibits a 150% enhancement in transconductance and a 60% reduction in variability compared to conventional spin-coated organic electrochemical transistors. The investigation further illustrates the potential impact of the technique on voltage spike-rate hardware classification in bio-signal sorting applications [108]. In another example, researchers have shown that the dendritic growth of PEDOT:PSS-based fibres using alternating current (AC) electropolymerization facilitates structural plasticity during network formation. This strategy adheres to Hebbian principles and provides topologies that enhance computational performance through sparse synaptic connectivity for addressing complicated tasks. The approach demonstrates an enhancement of up to 61% in network sparsity for classification and 50% for signal reconstruction tasks in simulations [109].

Organic computational materials are also promising candidates used for artificial neural networks due to their neuromorphic performance. Research has concentrated on creating hardware accelerator systems for in-memory computing, which minimizes energy-intensive processes, viz. multiply-accumulate operations [110–113]. These systems have been utilized in various applications, e.g., partial differential-equation solvers, long-term and short-term memory networks, and hardware convolutional neural networks. Organic artificial synapses represent intriguing options for artificial neural networks owing to their low-voltage functionality, analogue tuning, and linear programming capabilities (Fig. 5c). However, traditional organic neuromorphic devices demonstrate device-to-device variability, posing challenges when expanding to larger arrays. Nevertheless, the implementation of analogue tuning, expansive conductance windows, and resilient neural network operations alleviate the necessity for fault tolerance and minimal variability in conventional devices. For instance, recent works have revealed that the unique structural and electrical characteristics of one-dimensional covalent organic framework (COF) films, e.g., COF-4,4′-methylenedianiline (MDA) and COF-4,4′-oxydianiline (ODA), provides benefits for multilevel resistive switching, which is a crucial aspect of neuromorphic computing applications. The incorporation of a TiO_2_ layer onto the COF-ODA film generates a built-in electric field between the COF-TiO_2_ interfaces, illustrating the potential of COFs as a platform for developing organic neuromorphic devices with tunable resistive states. The one-dimensional nanochannels of the COF structures facilitated the effective modulation of electrical conductance, enabling precise control of synaptic weights in neuromorphic circuits. This work further investigated the capability of COF-based devices to achieve energy-efficient and high-density neuromorphic systems [114].

Solid electrolytes composed of ionic liquids within polymer matrices are appropriate for the fabrication of circuits and arrays alongside intricate peripheral electronics. A problem is the development of a patternable solid electrolyte to facilitate the upscaling of traditional crossbar arrays and to mitigate current sneak pathways and device-to-device crosstalk during read–write inference processes. To attain optimal energy gains, parallel updating has to be further employed, necessitating an innovative amalgamation of block writing, as only specific device combinations can be addressed separately. This necessitates a collaborative endeavour involving optimized peripheral electronic circuit design and specialized software tools for device access [71, 115, 116]. For example, researchers have shown that PEDOT:PSS formulation-based neuromorphic devices are able to perform both analogue computation and memory functions within the same device by altering its conductance. The solid-state formulation enables independent modulation of the organic neuromorphic device, hence mitigating write sneak-path problems. The organic neuromorphic device’s conductance exhibits exponential relaxation, with a measured time constant of 2.9 h. The estimated energy efficiency of the organic neuromorphic device is 0.1 pJ per multiply–accumulate operation. A 3 × 3 neuromorphic crossbar array has successfully demonstrated edge detection and blurring on a 128 × 64-pixel image [113].

The backpropagation algorithm utilizing gradient descent is the predominant algorithm in deep learning and neural networks [117–119]. However, implementing this in traditional hardware is difficult due to the requirement for storing weights’ partial derivatives. Alternative methodologies, e.g., training spiking neural networks or evolutionary processes, are advantageous for low-power organic neuromorphic devices that are readily adjustable. For instance, recent work has shown an organic spiking neural network (SNN) model and circuit implementation termed flex-SNN, developed for real-time data processing and minimizing communication costs in flexible sensor systems. The flex-SNN model comprises an input layer, excitatory neurons, and a fully connected synaptic matrix. The synapses consist of insulating and contact layers for organic thin-film transistors, enabling the compact fabrication of the flex-SNN circuit on flexible substrates. The flex-SNN circuit is scalable owing to the configurability of the synaptic matrix through metal wire crossings. The flex-SNN circuit achieves an inference accuracy of 97.0% in classifying 0 and 1 s inside the Modified National Institute of Standards and Technology dataset [120].

Organic neuromorphic materials are also utilized for sensorimotor learning and environmental intelligence. Sensorimotor integration constitutes a type of associative learning that uses external stimuli to adjust to a dynamic environment and enhance the efficacy of a system exhibiting embodied intelligence [121–126]. This approach operates locally on an organic neuromorphic circuit in real time and closed loop, subjecting the circuit to ambient inputs through iterative interaction. A robot in a maze is able to progressively learn to correlate navigation cues with turning decisions at intersections and successfully exit the maze through sensorimotor integration. For example, researchers have demonstrated the usage of organic electrochemical transistors to navigate a small robot through a maze. A semiconducting polymer, poly(2-(3,3′-bis(2-(2-(2-methoxyethoxy)ethoxy)ethoxy)-[2,2′-bithiophen]-5-yl)thieno[3,2-b]thiophene), functions as the active-channel material in organic electrochemical transistors. The neuromorphic circuit exhibits energy-efficient and reliable learning capabilities facilitated by the mixed ionic-electronic conduction of the organic semiconductor. The work showcases unprecedented proficiency in organic neuromorphics for effective local and decentralized learning [127].

Organic neuromorphic materials and devices greatly enhance localized and distributed learning as well as sensorimotor regulation [17, 128]. Decentralizing these attributes and employing flexible, self-healable polymers result in more resilient systems with enduring intelligence. Local training and learning can be enhanced through multimodalities by including integrated sensors and stimuli-responsive materials used for retinal systems, haptic technology, and thermoelectric modules. On-chip biochemical learning methodologies utilizing actual neurotransmitters in robotics further transcend conventional digital reinforcement learning techniques. For instance, recent investigations have revealed an organometal halide perovskite-based p-type–intrinsic–n-type optoelectronic synapse that exhibits both excitatory and inhibitory light-mediated synaptic functions. The optoelectronic synapse demonstrates a distinct response to visible light at multiple wavelengths, e.g., 435, 545, and 700 nm. An artificial visual reflex arc is generated to integrate perception, processing, and actuation of light signals, thereby mimicking a pupil reflex that is adaptively controlled by different muscles. This technique exemplifies the emulation of the sophisticated functioning of human sensory systems [129].

Organic neuromorphic devices augment environmental intelligence through widespread distribution [130, 131]. Inexpensive biosensors have been used to assess plant nutrient levels and identify spatiotemporal patterns, enhancing decision-making in precision agriculture. Organic neuromorphic devices serve as a foundation for extensive sensing, processing, and pattern recognition, enabling the perception, action, and adaptation of large structures including skyscrapers. These multimodal organic neuromorphic devices provide real-time measurement and extraction of audio, mechanical, and optical patterns, providing critical insights and immediate control over environmental noise, mechanical integrity, or energy consumption. For example, recent studies have disclosed a low-voltage operating photosynaptic transistor using the organic semiconductor poly(2,5-bis(3-alkylthiophen-2-yl) thieno[3,2-b] thiophene) (PBTTT-C14). Basic neurobiological synapse-like behaviours, e.g., excitatory post-synaptic current (EPSC), pair-pulse facilitation (PPF), short-term plasticity (STP), long-term plasticity (LTP), and learning–forgetting–memorizing dynamics analogous to the human brain, are demonstrated. The photoresponse characteristics, viz. photoresponsivity (R), detectivity (D*), and photo-to-dark current ratio, are estimated and analysed. The devices exhibit the transition from STP to LTP with varying initial photoexcitations. The researchers attribute the brain-like behaviour of these photosynaptic devices to the interfacial charge-trapping phenomenon occurring at the interface of the semiconducting channel and dielectric layer. The work further investigates the impact of interfacial traps on photosynaptic behaviour by photocurrent measurements at different light intensities. These devices display human emotion and mood swing-dependent learning and memory behaviours. These investigations illustrate the photosynaptic behaviour using an organic field-effect transistor, which can be tuned by modifying trap density at the semiconductor-dielectric interface through a self-assembled monolayer [132].

### Neuromorphic Bioelectronics and Biohybrid Systems

Apart from using organic computational materials for neuromorphic computing, organic neuromorphic devices and their arrays have the capability to engage or interface with biological systems, e.g., neuron-like cells (Fig. 5d, e) [133–139]. These biohybrid systems process biological information and encode cellular signals through electrical and electrochemical transduction mechanisms. The elevated sensitivity and selectivity of organic biosensing interfaces have facilitated the creation of biohybrid synapses utilizing a single neurotransmitter, alongside the collective and electrochemical manipulation of multiple neurotransmitters. For example, researchers have shown that organic neuromorphic devices using various PEDOT:PSS formulations with diverse geometries are able to interface with gel electrolytes loaded with a neurotransmitter to mimic brain-chip interfacing. Short-term plasticity and neurotransmitter-mediated long-term plasticity have been exhibited in conjunction with different gel electrolytes. The electrolytes and PEDOT:PSS formulations serve as gate and channel materials for diffusion and trapping of cations, hence modulating the transistor channels’ conductance. Researchers demonstrated that paired-pulse facilitation is attainable in electrochemical organic neuromorphic devices using PEDOT:PSS blend materials, whereas long-term plasticity can be achieved with a tissue-like soft electrolyte, e.g., agarose gel electrolyte, on spin-coated organic neuromorphic devices [140].

Hybrid systems, activated by biological signalling, also facilitate functional regulation and actuation of synaptic molecules, biological membranes, cell layers, tissues, organs, and living creatures. These systems enable two-way communication between artificial and natural realms, monitoring biological activities and providing stimulation as needed. Seamless integration and transparent interfaces between host biological systems and artificial neurons ensure long-term mechanical stability. Stretchable and flexible materials are optimal for device support and electroactive applications. By incorporating organic semiconductors with tissue-like materials, the Young’s modulus mismatch between the target tissue and the organic neuromorphic device can be significantly reduced. Neuromorphic systems should be appealing to cells and tissues, fostering enhanced interaction, and more effective signal transmission. Biohybrid neuromorphic systems provide in situ multimodal sensing and computation via iontronics, electrophysiology, electrochemistry, and optoelectronics, enabling real-time monitoring and processing of bio-signals from the human body [141–144]. For instance, researchers have demonstrated an efficient organic electrochemical neuron (OECN) with minimized footprint (< 37 mm^2^), using high-performance vertical-OECN complementary circuitry enabled by a sophisticated n-type polymer to achieve balanced p-/n-type OECN functionality. The OECN exhibits exceptional neuronal characteristics, capable of generating spikes with a highly adjustable firing-frequency range of 0.13 to 147.10 Hz. The researchers employ this capability to develop a neuromorphic perception system that integrates mechanical sensors with the OECN and incorporates an artificial synapse for tactile perception. The system successfully encodes tactile stimuli into frequency-dependent spikes, which are then converted into post-synaptic responses. This bio-inspired design exhibits significant potential to enhance cyborg and neuromorphic systems by endowing them with perceptive capabilities [145]. Nevertheless, closed-loop bidirectional communication, mimicking the human brain’s connection and learning characteristics, is lacking due to incomplete computational capabilities in organic interfaces. Techniques, e.g., brain wave classification and spike sorting, are utilized to process bio-signals. An innovative design combines organic neuromorphic devices as functional ionic-electronic transducing devices, with inorganic high-throughput devices for bio-signal treatment and information processing. This helps in prosthetics maintenance and operation, enhancing and restoring specific biological functions in the case of organ loss. For instance, recent works have disclosed that organic electrochemical transistors utilizing PEDOT:PSS have been employed for their ionic-to-electronic signal transduction and biocompatibility. A feedback-loop control is utilized to bidirectionally modulate the organic semiconductor using hydrogen peroxide, facilitating the partial recovery of the PEDOT:PSS doping level following its conditioning from neurotransmitter oxidation. The computational capabilities of the closed-loop system have been demonstrated in an organic circuit, in which the artificial ‘synaptic weights’, influenced by the PEDOT:PSS conductance, have been controlled to alter the circuit’s electrical dynamics. Furthermore, the robotic hand’s motors are connected using a neurotransmitter-mediated organic electrochemical transistor to regulate the hand’s opening and closing [146].

### Neuroscience

Beyond achieving neuromorphic bioelectronics and biohybrid systems based on organic neuromorphic devices, organic neuromorphic materials are utilized to monitor and manipulate large-scale cerebral processes with specificity to cell types for prolonged periods, spanning months and years. These qualities are essential for comprehending intricate neural population dynamics throughout development, learning, memory, and ageing (Fig. 5f) [147–152]. Extensive in vivo recording is crucial for delineating the functional connectomics of the brain, demonstrating the interconnections and coordination across various regions. By pinpointing specific neuronal groups and essential brain regions, brain–computer interface applications are able to precisely interpret brain states and direct behaviours [57, 153, 154]. For example, researchers have disclosed a high-density, ultraflexible organic electrochemical transistor array specifically designed for high-resolution electrocorticogram (ECoG) signal recording. The array includes vertically stacked source and drain electrodes, consisting of 1024 channels in a compact design, with a thickness of approximately 4.2 μm, and achieving a density of 10,000 transistors per square centimetre. A 16 × 16 segment of the 1024-channel array was utilized to map whisker-related signals in a mouse model, effectively identifying neural activities in response to tactile stimulation. Furthermore, it exhibits significant mechanical compliance and long-term stability, maintaining efficacy for three months post-implantation and beyond. The ultraflexible organic electrochemical transistor array, characterized by its excellent resolution and durability, is poised to enhance the monitoring and understanding of neural dynamics across a broad spatiotemporal scale [155].

Organic neuromorphic materials have also been employed to investigate biological processes, e.g., neural representational drift, which alters the activity of individual neurons in response to sensory stimuli or behavioural outputs over time [156–159]. These organic neuromorphic materials amalgamate cellular gene expression and connectivity data with single-cell electrophysiology, facilitating multimodal machine learning algorithms to deduce cell-type identity from brain recordings for enhanced brain-state decoding, identify biomarkers for the treatment of neurological disorders, and discern neuronal subtypes for precise recording and modulation. For instance, recent studies have shown that a conductive PEDOT:PSS polymer–hydrogel electroencephalogram electrode exhibits long-term stability and decreased electrode–skin interfacial impedance, maintaining a lower impedance value than gel-based electrodes for 29 days. This technology enables the creation of electroencephalogram-based long-term and wearable brain-computer interfaces. This designed electrode was utilized in a wireless single-channel electroencephalogram device to monitor variations in alpha rhythms during open and closed eye conditions. The engineered electrodes demonstrated the ability to detect oscillatory rhythms in motor imagery protocols and low-frequency time-locked event-related potentials from healthy subjects, exhibiting performance that is comparable or higher than that of gel-based electrodes. This work illustrates the usage of designed electrodes in online brain–computer interface-based functional electrical stimulation, which may be advantageous for post-stroke rehabilitation [160].

## Outlook and Conclusions

Organic computational materials have the potential to be utilized in neuromorphic computing due to their biocompatibility, low cost, excellent tunability, and low-energy switching. They offer access to a wide range of material states and electrical conductance or optical reflectivity, rendering them ideal for hardware-based vector–matrix multiplication and forward-inference neural networks. Customizing the mechanical, chemical, and electrical properties of organic compounds generates novel materials with low-energy switching capabilities, linear switching behaviour, and long-term stability, rendering them adaptable for various physical configurations. However, many open-ended questions about switching mechanisms remain. The success and commercialization of organic neuromorphic devices depend on its development, attainable through methodical studies of switching mechanisms. A comprehensive understanding of physics and simultaneous presence of various switching mechanisms is also required. The usage of appropriate computational and characterization tools is also essential.

Despite recent achievement, further research is required to overcome limitations in organic neuromorphic elements. Efforts should be undertaken to enhance state retention, minimize read and write noise, and implement encapsulation using protective polymer-based materials, e.g., Parylene or PDMS, to bolster cycling endurance and device stability for prolonged functionality.

High-quality manufacturing facilities additionally improve neuromorphic arrays, but they face issues with device reproducibility, especially when traditional devices are downsized to less than 1 µm^2^. This leads to array failure, and further refinement and research are needed. Nevertheless, the extensive conductance range of redox-coupled devices, e.g., PEI/PEDOT:PSS-based devices, establishes a tolerable range for suboptimal devices generated within the same array, which, when integrated with linear symmetric switching through optimized pulsed write schemes, could result in adequate array performance.

The development of a traditional efficient all-organic neuromorphic system is also hindered by the absence of mapping of input signals to output signals and an activation function. To enhance their versatility and attractiveness, it is crucial to incorporate organic tunable neuromorphic devices into inorganic or other conventional CMOS applications. For example, organic neuromorphic devices can be integrated into CMOS technology using organic computational materials, e.g., PEDOT:PSS, to create synaptic-like devices that interface directly with standard CMOS transistors. Further developments and exploration are required to achieve additional integration into the back-end-of-the-line processing, involving new materials that withstand high temperatures, e.g., in the 620–670 K temperature range. Highly effective inorganic–organic neuromorphic systems that leverages both technologies provide strong incentives for this endeavour. Organic computational materials can be useful for situations requiring minimal energy consumption and specialized local functionality, operating harmoniously with inorganic CMOS-based neuromorphic arrays.

The understanding of switching speed, a crucial aspect during training, is also limited. To improve it, researchers have to investigate the fundamental mechanisms of device operation and their materials’ reliance. A precise physical model, e.g., drift–diffusion models, of these devices is required, which enables accurate simulation of device behaviour and expedite design feedback loops. This would be highly beneficial in this domain.

The limited understanding of biological processes further presents an obstacle to advancement, as it leads to uncertainty in the correlation between these phenomena and the characteristics of organic neuromorphic materials and devices. The integration of organic neuromorphic devices, e.g., PEDOT:PSS devices, and supported lipid bilayers, viz. 1-palmitoyl-2-oleoyl-glycero-3-phosphocholine (POPC), with human activity has the potential to reveal new dimensions of interaction, aiding humans in areas, e.g., robotics, mixed or virtual reality, point-of-care diagnostics, bioelectronics, personalized medicine, environmental intelligence, and scientific and personal computing. These interaction dimensions create new opportunities for the advancement of techniques in managing and understanding biology, leading to unforeseen outcomes in the future. Regeneration, tissue engineering, neuromodulation, and biocomputing are currently entering a new phase of investigation, requiring the development of new tools for more natural interaction with biological systems. Biohybrid systems collaborate with humankind in a synergistic manner, similar to how animal domestication occurred in the past several millennia. However, ethical considerations arise when considering the enhancement of physical capabilities or sensory functions, as these technologies may be more readily available to certain socioeconomic groups. The management of intellectual property in human-made biohybrid systems that perform biocomputing is an uncharted domain.

In the future, organic neuromorphic devices, characterized by low-temperature processing methodologies and distinctive weight updating processes, could provide benefits, e.g., the facilitation of economical, disposable lab-on-chips and the eradication of sneak currents, for intelligent point-of-care systems. These advancements could result in improved hybrid organic and biological functionalities, including customizable local control of prosthetics, robotic skin, biosensor networks, and trainable and adaptive brain–computer interfaces. These improvements could also facilitate the creation of sophisticated robotic epidermis and biosensor networks.

## Data Availability

The authors declare that all data supporting the findings of this study are available within the article. Other data are available from the corresponding authors upon reasonable request.
